# Nongaussian Intravoxel Incoherent Motion Diffusion Weighted and Fast Exchange Regime Dynamic Contrast-Enhanced-MRI of Nasopharyngeal Carcinoma: Preliminary Study for Predicting Locoregional Failure

**DOI:** 10.3390/cancers13051128

**Published:** 2021-03-06

**Authors:** Ramesh Paudyal, Linda Chen, Jung Hun Oh, Kaveh Zakeri, Vaios Hatzoglou, C. Jillian Tsai, Nancy Lee, Amita Shukla-Dave

**Affiliations:** 1 Department of Medical Physics, Memorial Sloan Kettering Cancer Center, New York, NY 10065, USA; paudyalr@mskcc.org (R.P.); OhJ@mskcc.org (J.H.O.); 2 Department of Radiation Oncology, Memorial Sloan Kettering Cancer Center, New York, NY 10065, USA; chenl1@mskcc.org (L.C.); ZakeriK@mskcc.org (K.Z.); tsaic@mskcc.org (C.J.T.); leen2@mskcc.org (N.L.); 3 Department of Radiology, Memorial Sloan Kettering Cancer Center, New York, NY 10065, USA; HatzoglV@mskcc.org

**Keywords:** diffusion weighted, gaussian and non-gaussian, kurtosis, dynamic contrast-enhanced, fast exchange regime, permeability, metabolic activity

## Abstract

**Simple Summary:**

Pre-treatment (TX) prediction of the risk of locoregional failure (LRF) will allow for TX individualization in patients with nasopharyngeal carcinoma (NPC). The aim of the present study was to identify whether the quantitative metrics from pre-TX non-Gaussian intravoxel incoherent motion (NGIVIM) diffusion weighted (DW-) and fast exchange regime (FXR) dynamic contrast enhanced can predict patients with LRF in NPC. Cumulative incidence (CI) analysis and Fine-Gray (FG) proportional subhazards modeling were conducted in a sample of 29 NPC patients considering death as a competing risk. NGIVIM and FXR derived quantitative metric values from primary tumors classified the patients with and without LRF in NPC. The CI analysis and FG modeling results suggest that the quantitative metrics obtained from DW- and DCE-MRI may improve LRF patients’ prediction in NPC.

**Abstract:**

The aim of the present study was to identify whether the quantitative metrics from pre-treatment (TX) non-Gaussian intravoxel incoherent motion (NGIVIM) diffusion weighted (DW-) and fast exchange regime (FXR) dynamic contrast enhanced (DCE)-MRI can predict patients with locoregional failure (LRF) in nasopharyngeal carcinoma (NPC). Twenty-nine NPC patients underwent pre-TX DW- and DCE-MRI on a 3T MR scanner. DW imaging data from primary tumors were fitted to monoexponential (ADC) and NGIVIM (*D*, D*, f, and *K*) models. The metrics K^trans^, v_e_, and τ_i_ were estimated using the FXR model. Cumulative incidence (CI) analysis and Fine-Gray (FG) modeling were performed considering death as a competing risk. Mean v_e_ values were significantly different between patients with and without LRF (*p* = 0.03). Mean f values showed a trend towards the difference between the groups (*p* = 0.08). Histograms exhibited inter primary tumor heterogeneity. The CI curves showed significant differences for the dichotomized cutoff value of ADC ≤ 0.68 × 10^−3^ (mm^2^/s), *D* ≤ 0.74 × 10^−3^ (mm^2^/s), and f ≤ 0.18 (*p* < 0.05). τ_i_ ≤ 0.89 (s) cutoff value showed borderline significance (*p* = 0.098). FG’s modeling showed a significant difference for the *K* cutoff value of ≤0.86 (*p* = 0.034). Results suggest that the role of pre-TX NGIVIM DW- and FXR DCE-MRI-derived metrics for predicting LRF in NPC than alone.

## 1. Introduction

Nasopharyngeal carcinoma (NPC) arises from the mucosal lining of the nasopharynx and is associated with the Epstein-Barr virus (EBV) [1]. The current standard of care for locally advanced NPC is definitive chemoradiation with either neoadjuvant or adjuvant chemotherapy [2,3]. The role of chemotherapy regimens, proton therapy, and EBV-directed adjuvant therapy is still being evaluated [4,5,6]. Intensity-modulated radiotherapy (RT) has allowed precise targeting as well as a reduction in toxicity through sparing of normal tissue [7,8,9,10]. Due to the deep anatomic location and proximity to critical structures, treatment (TX) is still associated with toxicities [11]. Noninvasive imaging modalities, such as MR imaging, computed tomography (CT), and positron emission tomography (PET/CT), have shown potential for the management of NPC [12]. The imaging biomarkers that can predict early TX response and local recurrence are needed to personalize therapy.

Quantitative diffusion-weighted magnetic resonance imaging (DW-MRI) measures the random translational motion of water molecules in tissue [13]. The pre-TX apparent diffusion coefficient (ADC) has been shown to have promise for tumor staging and predicting TX response in NPC [14,15]. Chen et al. reported that the pre-TX mean ADC values of primary tumors were not significantly different between the responder group (RG) and non-responder group (NRG) in NPC [15]. Law et al. found that the pre-TX skewness ADC value of the primary tumors was a predictor of locoregional failure (LRF) in NPC [16]. The quantitative imaging (QI) metrics derived from the intravoxel incoherent motion (IVIM) model [13] demonstrated their ability to capture the chemo-RT response in metastatic lymph nodes of the head and neck (HN) cancer [17,18]. Lu et al. incorporated the Kurtosis coefficient (*K*) into the IVIM model [19], which accounts for the deviations of diffusion from Gaussianity (non-Gaussian [NG] IVIM) without a contrast agent (CA), to characterize the hindered and restricted distribution of water molecules simultaneously in HN cancer [13,20]. The clinical application showed that the *K* (%) values in the first week of chemo-RT were significantly different between the complete response and residual patients of human papillomavirus-positive oropharyngeal cancer who were treated with dose de-escalation, 70 to 30 Gy [21,22].

T_1_-weighted dynamic contrast-enhanced (DCE)-MRI acquired before, during, and after a bolus administration of CA [23] can be modeled with the three-parameter Tofts model, which assumes a fast water exchange between tissue compartments. The previous study results have shown that volume transfer constant (K^trans^) was related to NPC’s clinical-stage [24]. The skewness of K^trans^ was the strongest predictor of progression-free and overall survival in stage IV HN cancers [25]. The fast exchange regime (FXR) model, accounting for water exchange between intracellular space (ICS) and extracellular space (EES) [26], provides estimates of the mean lifetime of intracellular water protons (τ_i_) in addition to K^trans^ and volume fraction of the extravascular extracellular space (EES) [v_e_]. The FXR pre-TX K^trans^ has been shown to have the ability to predict response to chemo-RT in HN cancer [22,27]. Chawla et al. have reported that τ_i_, an index of cell metabolic activity, was higher in patients with the most prolonged overall survival in HN cancer [28].

The integration of quantitative DW- and DCE-MRI techniques offer insight into tumor cellularity, vessel permeability, and metabolic activity. These functional MRI biomarkers correlating with disease state may help predict early TX failure that may allow selecting a personalized TX strategy in NPC. Using the pre-TX metric value, the different advanced statistical techniques based on a competing risks model have been used to predict the outcome, such as locoregional failure [29,30].

The aim of the present study was to identify whether quantitative metrics obtained with pre-TX NGIVIM DW- and FXR DCE-MRI can predict patients with LRF in NPC. Cumulative incidence (CI) analysis and Fine and Gray (FG) modeling [31], which estimates subdistribution hazard ratios (SDRs), were performed based on a competing risks model.

## 2. Materials and Methods

### 2.1. Patients

The institutional review board approved this retrospective study, and written informed consent was obtained from all eligible NPC patients prior to the pre-TX MRI study. Between November–June 2014 and September 2016, a total of 29 NPC patients treated with definitive chemoradiation (70 Gy) who had pre-TX DWI and DCE-MRI were included. Follow-up data and imaging were reviewed. LRF was defined as a persistent or recurrent HN disease.

### 2.2. MRI Data Acquisition

MRI scans were performed on a Philips 3T scanner (Ingenia; Philips Healthcare, Netherlands) using a neurovascular phased-array coil [32]. The standard MR acquisition comprised multi-planar T_2_-weighted (T_2_w) (repetition time [TR] = 4000 ms, echo time [TE] = 80 ms, number of averages [NA] = 2, and number of slices [NS] = 40, matrix = 256 × 256, slice thickness = 5 mm, field of view [FOV] = 20–24 cm) and T1w imaging (TR = 600 ms, TE = 8 ms, NA = 2, NS = 40, slice thickness = 5.0 mm; matrix = 256 × 256, FOV = 20–24 cm). T_1_w and T_2_w acquisitions were followed by DW- and DCE-MRI, and the total acquisition time was approximately 30 min.

### 2.3. DWI Data Acquisition

The multiple b-values DW-MRI images were acquired using a single-shot echo-planar imaging sequence (TR  =  4000 ms, TE = minimum [80 ms], NA = 2, matrix  =  128 × 128, FOV  =  20–24 cm, NS  =  8–10, slice thickness  =  5 mm, and 10 b-values [b = 0, 20, 50, 80, 200, 300, 500, 800, 1500, 2000 s/mm^2^]). The total acquisition time was approximately 5 min.

### 2.4. DCE Data Acquisition

T_1_w dynamic images were acquired using a fast three-dimensional spoiled gradient -recalled sequence (TR = 7 ms; TE = 2.7 ms, slice thickness = 5 mm, flip angle (FA) = 15°, FOV = 20–24 cm, NS = 8–10, matrix 256  ×  128 that was zero-filled to 256 × 256 during image reconstruction). A total of 50 phases were acquired before, during, and after a bolus injection of 0.1 mmol/kg Gd-based CA, Gadobutrol (Gadavist, Bayer Health Care), delivered through an antecubital vein catheter at 2 cc/sec, followed by a saline flush using an MR-compatible programmable power injector (Spectris; Medrad, Indianola, PA, USA). The temporal resolution ranged from 7.2 to 8.96 sec/image, and the total acquisition time was ≤8.0 min.

The precontrast T_1w_ images were acquired prior to the dynamic series with the same MR acquisition parameters as mentioned above using three different FAs of 30°, 15°, and 5° for T_10_ mapping.

### 2.5. DWI Data Analysis

The multiple b-value DW-MRI data were fitted to the (a) monoexponential Equation (1) and (b) bi-exponential (NGIVIM) model Equation (2) as follows [13,33]

S(b) = S_0_*e*^−*b*×ADC^, (1)

(2) $$S(b)=S_{0}\;[f\;e^{-b\times D^{*}}\;+\;(1\;-\;f)\;e^{-b\times D+\frac{1}{6}K{\left(b\times D\right)}^{2}}],$$

where S(b) and S_0_ are the signals with and without diffusion weighting gradient factor, b (s/mm^2^), ADC (mm^2^/s), *D* (mm^2^/s), and D*(mm^2^/s) are the apparent, true, and pseudo-diffusion coefficients (mm^2^/s), respectively, f is the perfusion fraction, and *K* is the kurtosis coefficient (unitless).

### 2.6. Fast Exchange Regime Pharmacokinetic Analysis

The tissue relaxation, *R*_1*t*_ (*R*_1*t*_ = 1/*T*_1*t*_) Equation (3), and EES relaxation, *R*_1*e*_, Equation (4) for fast exchange limit, are given as follows [23,34]

*R*_1*t*_ = *R*_10_ + r_1_ C_t_(t), (3)

*R*_1*e*_ = *R*_10*e*_ + r_1_ Ce(t), (4)

where *R*_10_ and *R*_10*e*_ are the pre-contrast relaxation rates of the tissue and EES, respectively, r_1_ is the longitudinal relaxivity of CA (r_1_ = 4.0 mM^−1^s^−1^). C_t_(t) and C_e_(t) are the CA concentration in tissue and EES with time, respectively. The C_t_ and C_e_ are related as: C_t_ = v_e_ × C_e_, where v_e_ is the volume fraction of EES.

The standard Tofts model C_t_(t) is given by Equation (5) [23].

(5) $$C_{t}(t)\;=\;K^{\mathrm{trans}}\int_{0}^{t}C_{p}e^{-k_{ep}\left(t-\tau \right)}d\tau ,$$

where K^trans^ is the volume transfer constant for CA, v_e_ is the volume fraction of EES, *k_ep_* (*k_ep_* = K^trans^/v_e_) represents the rate constant of CA transport from the vascular space to EES, Cp(t) is the time course of plasma CA concentration (called arterial input function [AIF]).

The fast exchange regime model (FXR), the shutter speed model, incorporates the equilibrium two-site water exchange (2SX) between ICS and EES (i.e., transcytolemmal) to analyze the DCE data. The observable R_1_ is derived by solving Bloch McConnell’s equation for the 2SX model [35], a variant form of the three-site two water exchange model [36]. One of the eigenvalues of the 2SX is the observable *R*_1*t*_(*t*) for FXR and is given by Equation (6) [37]

(6) $$R_{1}\left(t\right)\;=\;\frac{1}{2}\left[\left(R_{1i}++k_{ie}+R_{1e}+k_{ei}\right)-\sqrt{{\left(R_{1i}++k_{ie}-R_{1e}-k_{ei}\right)}^{2}}\right],$$

where *R*_1*i*_ and *R*_10*e*_ are the precontrast relaxation rates of ICS and ESS, and *k_ie_* (*k_ie_* = 1/τ_i_) and *k_ei_* are the rates of water exchange from the ICS to EES and vice versa. The FXR provides estimates of K^trans^ (min^−1^), v_e_, and τ_i_ (s).

### 2.7. Regions of Interest (ROIs) Analysis

ROIs were delineated by radiation oncologists on the DW image (b = 0 s/mm^2^) and late phase of the T_1w_ dynamic images of primary tumors in NPC. The primary tumor volume was calculated from T_2_-weighted images as detailed elsewhere [32]. The ROI contouring was performed using Image J [38].

The pre-contrast T_1w_ FAs data were fitted for T_10_ mapping as detailed elsewhere [39]. The *R*_1_ data with time were fitted to Equation [6] using the AIF was extracted from the carotid artery [25].

The NGIVIM DW- and FXR DCE-MRI models were fitted using a nonlinear fitting algorithm as detailed elsewhere [20,37]. The post image processing and parametric map generation were performed using Quantitative Analysis Multi-Parametric Evaluation Routines (MRI-QAMPER) software, written in MATLAB™ (The MathWorks, Inc., Natick, MA, USA) [40,41].

### 2.8. Statistical Analysis

The pre-TX quantitative metrics values within the ROI were reported as the mean, standard deviation, skewness, and kurtosis. The histogram plots were generated for each metric value for visual examination of the intra/inter-tumor heterogeneity. The Wilcoxon rank-sum test was used for comparing the metric values between patients with and without LRF of NPC. Cumulative incidence analysis (CIA) was performed with dichotomized value for each metric based on Youden’s index [42] and tested for significance using Gray’s test. CI analysis considers death a competing risk factor that includes different events, such as a locoregional failure, but the interest lies in the first occurring event [43], which was performed. Competing risks regression was conducted using the Fine and Gray’s (FG) proportional sub hazards model to estimate subdistribution hazard ratios (SHRs) [31], and adjusted 95% confidence intervals were reported, considering death as a competing risk. Statistical significance was set at *p* < 0.05. All statistical computations were performed using the R version 4.0.3 software [44].

## 3. Results

### 3.1. Clinical

The median age was 43 (range: 21–67), and 83% of patients had stage 3 disease (American Joint Committee on Cancer (AJCC 8) [45]. Age, gender, stage, and EBV association for patients with and without LRF are summarized in Table 1.

The mean tumor volumes in patients with and without LRF were not significantly different (7.64 ± 7.24 cm^3^ vs. 9.15 ± 7.52 cm^3^, *p* = 0.4).

### 3.2. DWI Data Analysis

One of the 29 patients was excluded from DWI analysis due to image distortion and susceptibility artifact (Table 1).

**Figure 1:**
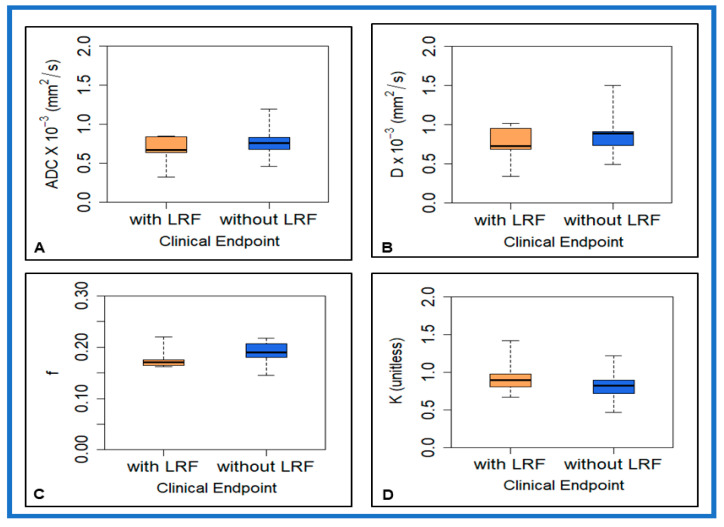
Box plot comparing the pre-treatment mean value of (**A**) apparent diffusion coefficient (ADC), (**B**) true diffusion coefficient (D), (**C**) perfusion fraction (f), and (**D**) kurtosis coefficient (*K*) between patients with and without locoregional failure in nasopharyngeal cancer. f showed a certain trend towards significance (*p* = 0.08). Boxes represent interquartile range. Whiskers represent range of all values. Horizontal line within box is median value.

Figure 1 shows a box plot for pre-TX ADC, *D*, f, and *K* values from primary tumors in patients with and without LRF of NPC.

Mean pre-TX f values showed borderline significance between patients with and without LRF in NPC (*p* = 0.08). Mean pre-TX ADC, *D*, D*, and *K* values showed a trend towards the difference but were not significantly different between the groups (*p* > 0.05). The mean SD of ADC and *D* values trended towards a considerable difference (*p* = 0.15 for ADC and *p* = 0.14 for *D*). The mean skewness *K* values showed the limit of significance between the groups (*p* = 0.16). Table 2 summarizes the ADC, NG-IVIM model derived *D*, D*, f, and *K* values (mean ± SD).

**Figure 2:**
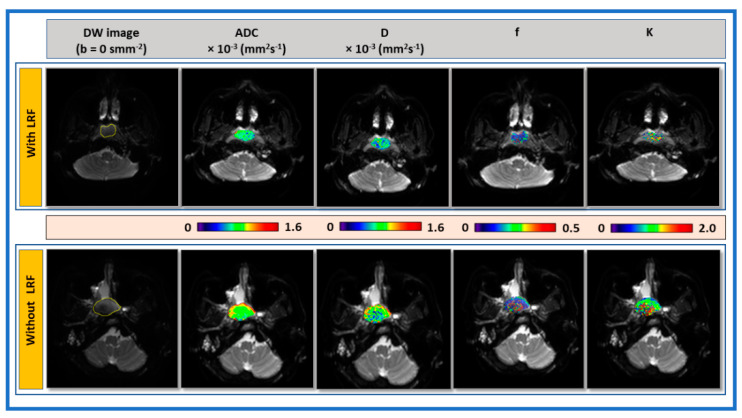
Representative pre-treatment (TX) diffusion-weighted (DW) images (b = 0 s/mm^2^) and parametric maps of apparent diffusion coefficient (ADC), true diffusion coefficient (*D*), perfusion fraction (f), and kurtosis coefficient (*K*) in patients with (48-year-old male) and without locoregional failure (LRF) (55-year-old male) of nasopharyngeal carcinoma (top and bottom). Primary tumor parametric maps are overlaid on pre-TX DW images (b = 0 s/mm^2^)

Figure 2 displays the representative pre-TX DW images (b = 0 s/mm^2^) with ROIs and parametric maps of ADC, *D*, f, and *K* overlaid on the DW image (b = 0 s/mm^2^) from primary tumors in patients with and without LRF of NPC.

**Figure 3:**
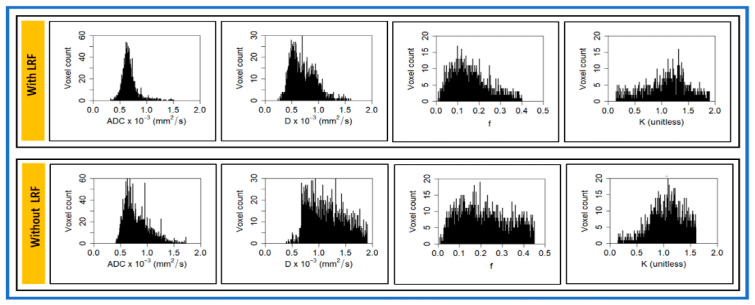
Representative voxel value distribution of apparent diffusion coefficient (ADC), true diffusion coefficient (*D*), perfusion fraction (f), and kurtosis coefficient (*K*) in patients with and without locoregional failure (LRF) of nasopharyngeal cancer (Figure 2). AD, *D*, and f values trended higher in a patient without LRF than compared with a patient with LRF. By contrast, *K* values trended higher in a patient with LRF compared with a patient without LRF. Histogram demonstrates distribution of each metric voxel value within ROI.

Histograms exhibit the distribution of metric values in the ROI. The representative histogram of ADC, *D*, f, and *K* shows the asymmetrical distribution of metric values in patients with and without LRF (Figure 3). ADC, *D*, and f values trended higher in a patient without LRF than with LRF. By contrast, *K* values trended higher with LRF than without LRF. The mean kurtosis of ADC (3.46 vs. 9.949), *D* (3.88 vs. 13.91), and f (1.58 vs. 2.20) values were lower in a patient without LRF than with LRF. By contrast, mean *K* values (1.26 vs. 1.12) were slightly higher without LRF.

### 3.3. FXR DCE-MRI Analysis

**Figure 4:**
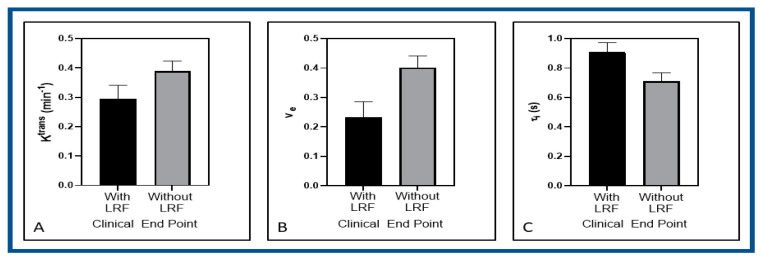
Bar plot comparing the mean value of (**A**) volume transfer constant (K^trans^), (**B**) extravascular extracellular space volume fraction (v_e_), and (**C**) mean lifetime of intracellular water protons (τ_i_) from fast exchange regime model between patients with and without locoregional failure (LRF) of nasopharyngeal carcinoma. The metric v_e_ exhibited a significant difference between patients with and without LRF. K^trans^ and τ_i_ showed little significance (*p* = 0.14 for K^trans^ and *p* = 0.11 for τ_i_).

A total of 29 primary tumors DCE data from NPC patients were analyzed (Table 1). Figure 4 shows a bar plot for K^trans^, v_e_, and τ_i_ patients with and without LRF in NPC. The mean v_e_ value was significantly higher in patients without LRF than those with LRF of NPC (*p* = 0.03). K^trans^ and τ_i_ values trended towards significance between two groups (*p* = 0.14 for K^trans^ and *p* = 0.11 for τ_i_). Mean kurtosis of v_e_ and skewness of τ_i_ values showed a borderline significant difference between the groups (*p* = 0.11 for v_e_ and *p* = 0.09 for τ_i_). Table 3 shows the K^trans^, v_e_, and τ_i_ values (mean ± SD) from 29 NPC patients’ primary tumors.

**Figure 5:**
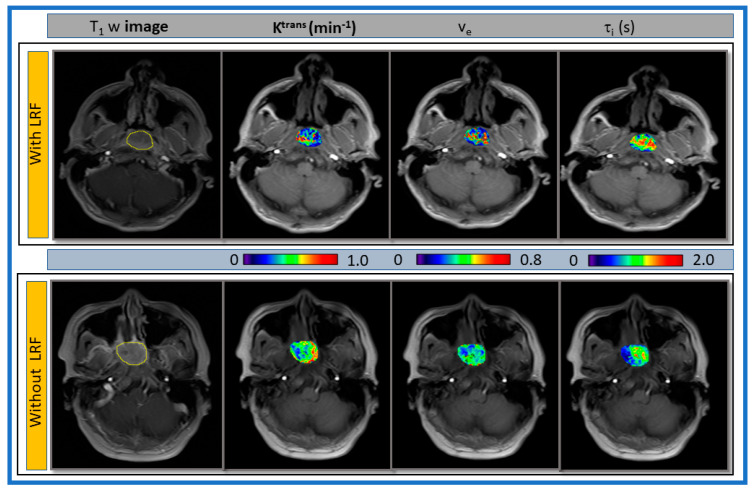
Representative pre-treatment (TX) T_1_w images from a late phase dynamic series from patients with (67-year-old female) and without locoregional failure (LRF) (55-year-old male) of nasopharyngeal cancer. Primary tumor parametric maps of volume transfer constant (K^trans^), extravascular extracellular space volume fraction (v_e_), and mean lifetime of intracellular water protons (τ_i_) overlaid on pre-TX T_1_w images.

Representative pre-TX T_1w_ MR images of a late phase dynamic series with ROIs and parametric maps of primary tumors K^trans^, v_e_, and τ_i_ overlaid on T_1_w MR images for a patient with and without LRF of NPC are displayed in Figure 5.

**Figure 6:**
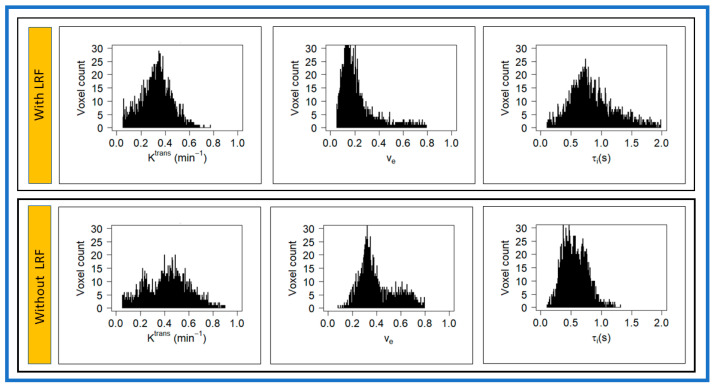
Representative voxel value distribution of volume transfer constant (Ktrans), extravascular extracellular space volume fraction (ve), and mean lifetime of intracellular water protons (τi) in a patient with and without LRF of nasopharyngeal cancer (Figure 4). Ktrans and ve values trended higher in a patient without LRF as compared with a patient with LRF. By contrast, τi values trended higher in a patient with LRF as compared with a patient without LRF.

The representative K^trans^, v_e_, and τ_i_ histograms generated from the voxel values for a patient with and without LRF of NPC are displayed in Figure 6. K^trans^ and v_e_ value trended higher in a patient without LRF than that with LRF. By contrast, τ_i_ values trended higher in a patient with LRF. The kurtosis of K^trans^ (9.6 vs. 3.18) values was higher in patients without LRF than with LRF. The metric τ_i_ kurtosis value (7.46 vs. 2.71) was higher with LRF than without LRF.

### 3.4. Survival Analysis: CIA and FG Proportional Subhazards Model

QI metrics value obtained from DW- (*n* = 28) and DCE-MRI (*n* = 29) data were used in a follow-up study. The follow-up periods ranged from 3 to 32 months (median: 17 months). The CIA, FG proportional subhazards model, and *p* values are listed in Table 4.

**Figure 7:**
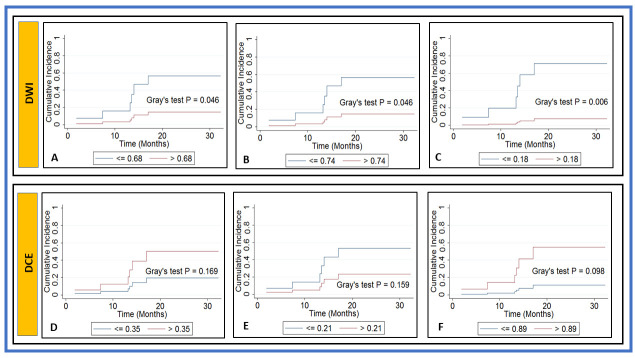
Cumulative incidence analysis based on locoregional failure (LRF) for pre-treatment (**A**) apparent diffusion coefficient (ADC [mm^2^/s]), (**B**) true diffusion coefficient (*D* [mm^2^/s]), (**C**) perfusion fraction (f), (**D**) volume transfer constant (K^trans^ [min^−1^]), (**E**) volume fraction of extravascular extracellular space (v_e_), and (**F**) mean lifetime of intracellular water protons (τ_i_ [s]). Gray’s test revealed a significant difference for ADC, *D*, and f (*p* < 0.05), and borderline significance for τ_i_ (s) (*p* = 0.098).

The CIA revealed that the two subgroups dichotomized with the cutoff value of ADC ≤ 0.68 × 10^−3^ (mm^2^/s) and *D* ≤ 0.74 × 10^−3^ (mm^2^/s) showed a statistically significant difference in the incidence of LRF (Gray’s test *p* = 0.046 for both). Additionally, the cutoff value of f ≤ 0.18 showed a significant difference in the incidence of LRF (Gray’s test *p* = 0.006), and the cutoff value of τ_i_ ≤ 0.89 (sec) had borderline significance (Gray’s test *p* = 0.098). No significant association was found for a cutoff value of D* ≤ 2.25×10^−3^ (mm^2^/s), *K* ≤ 0.86 (unitless), K^trans^ ≤ 0.35 (min^−1^), and v_e_ ≤ 0.21 with LRF (*p* > 0.05 for all. Comparative CIA curves are depicted in Figure 7.

FG analysis showed that the *K* values were significantly associated with LRF (SHR = 93.06, *p* = 0.034). The metric τ_i_ values showed borderline association in patients with LRF (SHR = 99.87 and *p* = 0.072).

## 4. Discussion

The present study evaluated the pre-TX DW- and DCE-MRI-derived quantitative imaging metrics’ ability to predict LRF in NPC. The differences of pre-TX ADC (14%), *D* (17%), *K* (13%), and f (7%) captured the varying tumor cellularity, vascularity, and microstructure between the patients with and without LRF. The change of pre-TX K^trans^ (32%), v_e_ (91%), and τ_i_ (22%) were able to characterize vessel perfusion/permeability, CA distribution space, and metabolic cell activity between patients with and without LRF. The cumulative incidence analysis (CIA) and Fine Gray modeling were performed to assess the incidence of LRF, considering death as competing risks.

ADC, *D*, and f cutoff values were significantly associated with LRF patients. Additionally, the FG model revealed that the metric *K* could be a predictor of outcomes. The histograms showed an asymmetrical distribution of metrics values with and without LRF, indicating inter-tumor heterogeneity. The results indicated that pre-TX quantitative metrics could be a useful prognostic marker for the prediction of LRF that will allow TX individualization in NPC [16,24]. The study findings were consistent with previous results, indicating a higher mean ADC value in patients without LRF than with LRF of NPC [15,16]. Tu et al. reported that the RG demonstrated a higher ADC, Dapp, and lower apparent kurtosis coefficient, K_app_, values compared with the NRG [46]. The present study is consistent with this finding. The metric *K* showed a higher sub-hazards ratio than ADC, *D*, f, and D*. In contrast, metrics ADC, *D*, and f are related to a cumulative incidence of LRF thank *K*.

Additionally, low K^trans^ and high τ_i_ values in patients with LRF are consistent with the previous results [27,28]. Higher pre-TX K^trans^ values exhibited an improved response to chemo-RT and prolonged survival [27,47] because the K^trans^ correlates with the proliferating cell density and micro-vessel density. Chawla et al. have reported that patients with high τ_i_ were associated with more prolonged overall survival than other groups in HN cancer [28]. The metric τ_i_ showed a higher sub-hazards ratio than K^trans^ and v_e_.

Histogram analysis shows the distribution of metrics values in the ROI that can directly represent Intra/inter tumor heterogeneity. The descriptive statistics represent the asymmetry of the distribution and the voxel-by-voxel value’s peakedness within the histogram. The metric values ADC, *D*, f, K^trans,^ and v_e_ of with LRF are leaning towards as compared to without LRF. In contrast, *K* and τ_i_ values are leaning toward higher values. The shift of metric value towards lower or higher may be associated with the extent of malignancy. Skewness and kurtosis of ADC and K^trans^ distributions, respectively, were used to predict TX response in HN cancers [16,48]. The present study showed a trend towards significantly higher skewness and kurtosis of ADC values in patients with LRF than those without LRF (Figure 4). By contrast, Law et al. have reported opposite results [16]. This discrepancy was possibly due to long-term follow-up and large sample size. The broader peaks towards higher values were seen in patients without LRF than those with LRF for ADC, *D*, D*, f, K^trans^, and v_e_. By contrast, *K* and τ_i_ values trended lower. The shape of the histogram revealed the tumor heterogeneity and microstructural differences in patients with and without LRF of NPC.

Despite progress in the management of locally advanced NPC, TX is still associated with significant toxicities [7,11]. Radiomic signatures derived from quantitative imaging features have the potential to guide clinical decision-making by identifying tumors at risk for treatment-resistance [49]. This has led to the exploration of CT, PET/CT, and MRI based radiomic nomograms in NPC, and while there is data to suggest that these models are comparable to TNM based staging symptoms for recurrence risk or EBV status, there remains discordance with regard to which imaging modalities and features are most prognostic and reproducible be-tween datasets [50,51,52].

The utilization of noninvasive DW- and DCE-MRI can be a valuable tool for the management of NPC patients because they reflect the physiological changes that occur at the cellular and metabolic levels in tumor tissue [15,28]. In the present study, *D*, *K*, K^trans^, and τ_i_ reflected the differences in tumor cellularity, the complexity of microstructure, vascularity, and metabolic activity in patients with and without LRF. Such findings are consistent with prior studies [16,27,28] and indicate that these metrics could be regarded as prognostic biomarkers in NPC. Additionally, a quantitative metrics map that displays tumor heterogeneity and significant regional change in TX responsiveness/resistance would allow for personalized TX planning and monitoring early TX response in NPC. Indeed, this could allow for personalization of the number of cycles of induction or adjuvant chemotherapy or identifying regions that require radiation dose escalation.

The present study has a few limitations. The patient cohort that led to a nonsignificant difference in quantitative metrics values between the two groups was relatively small. Evaluation in a larger cohort of NPC patients is warranted. The SE-EPI DW imaging sequence is generally insensitive to artifacts arising from bulk motions but suffers from severe geometric distortion and artifacts at tissue-air interfaces in the HN region due to magnetic susceptibility artifacts, especially at higher field strengths [53,54]. The temporal resolution, approximately 8 s, comprises the temporal resolution and spatial resolution to extract an arterial input function. The present study did not account for the B_1_ inhomogeneity correction associated with the flip angle. Moreover, while eligible NPC patients were prospectively enrolled pre-TX, the study did require completion of additional MRI imaging, and this may have unintentionally selected for patients with improved performance status who could complete the additional appointments. Thus, we are subject to the inherent limitations as a small single-institution study, and our findings will need to be evaluated in a larger multi-center cohort for validation.

## 5. Conclusions

The present study demonstrated that the pre-TX DW- and DCE-MRI derived QI metrics can identify diffusion and perfusion characteristics at the primary site, based on clinical follow-up in NPC. Knowing which patients fail early TX will help individualize care. A larger cohort is needed to ascertain the present findings further.

## Figures and Tables

**Table 1:** Patient characteristics.

Characteristics	Patients with LRF (*n* = 6)	Patients without LRF (*n* = 22)
Male/Female (%)	4/2 (14/7)	16/7 (55/24)
Age: median(range)	45 (21–64 years)	45 (21–64 years)
Stage III and IV (AJCC, %)	60/40	71/29
EBV-associated (%)	40	83

Note: AJCC: American Joint Committee on Cancer, EBV: Epstein-Barr virus, LRF: locoregional failure.

**Table 2:** ADC and NG-IVIM derived metric values.

Model	Metric	With LRF	Without LRF	*p*-Value
Monoexponential	ADC × 10^−3^ (mm^2^/s)	0.66 ± 0.19	0.76 ± 0.15	0.31
NG-IVIM	*D* × 10^−3^ (mm^2^/s)	0.74 ± 0.23	0.87 ± 0.22	0.31
	D* × 10^−3^ (mm^2^/s)	2.30 ± 0.25	2.40 ± 0.18	0.48
	f	0.17 ± 0.02	0.19 ± 0.02	0.08
	*K*	0.94 ± 0.25	0.82 ± 0.15	0.23

Note: LRF: locoregional failure.

**Table 3:** Summarizes the FXR DCE-MRI-derived metric values.

Metric	With LRF	Without LRF	*p*-Value
K^trans^ (min^−1^)	0.29 ± 0.11	0.39 ± 0.16	0.14
v_e_	0.23 ± 0.13	0.44 ± 0.21	0.03
τ_i_ (s)	0.91 ± 0.15	0.71 ± 0.27	0.11

Note: LRF: Locoregional failure.

**Table 4:** Survival Analysis.

Method	Parameter	Cumulative Incidence Analysis	Competing Risks Regression		
		Gray’s Test (*p*-Value)	Subdistribution Hazard Ratio (SHR)	*p*-Value	95% CI
DWI	ADC × 10^−3^ (mm^2^/s)	≤0.68 vs. >0.68 *p* = 0.046	0.03	0.17	0.00–4.37
	*D* × 10^−3^ (mm^2^/s)	≤0.74 vs. >0.74 *p* = 0.046	0.08	0.20	0.00–4.05
	D* × 10^−3^ (mm^2^/s)	≤2.25 vs. >2.25 *p* = 0.115	0.29	0.45	0.011–7.40
	f	≤0.18 vs. >0.18 *p* = 0.006	93.06	0.034	1.42–6082.28
	*K*	≤0.86 vs. >0.86 *p* = 0.226	0.00	0.14	0.0–34,096.0
DCE	K^trans ^ (min^−1^)	≤0.35 vs. >0.35 *p* = 0.169	1.02	0.98	0.03–33.17
	v_e_	≤0.21 vs. >0.21 *p* = 0.159	0.17	0.42	0.00–13.62
	τ_i_ (s)	≤0.89 vs. >0.89 0.098	99.87	0.07	0.66–15,080.66

## Data Availability

The data presented in this study will be provided upon reasonable request.
